# Teacher anger as a double-edged sword: Contrasting trait and emotional labor effects

**DOI:** 10.1007/s11031-023-10027-0

**Published:** 2023-05-16

**Authors:** Hui Wang, Ming Ming Chiu, Nathan C. Hall

**Affiliations:** 1 Department of Special Education and Counselling, Faculty of Education and Human Development, The Education University of Hong Kong, Tai Po, Hong Kong; 2 Assessment Research Center, The Education University of Hong Kong, Tai Po, Hong Kong; 3 Department of Educational and Counselling Psychology, McGill University, Montreal, Canada

**Keywords:** Teachers’ emotional labor, Teacher emotions, Anger, Teacher-student relationships, Daily diary investigation, Teacher-perceived student engagement

## Abstract

In contrast to teachers’ positive emotions, such as enjoyment and enthusiasm, teachers’ negative emotions and the regulation of negative emotions have received limited empirical attention. As the most commonly experienced negative emotion in teachers, anger has to date demonstrated mixed effects on teacher development. On the one hand, habitual experiences of anger (i.e., *trait anger*) exhaust teachers’ cognitive resources and impair pedagogical effectiveness, leading to poor student engagement. On the other hand, strategically expressing, faking, or hiding anger in daily, dynamic interactions with students can help teachers achieve instructional goals, foster student concentration, and facilitate student engagement. The current study adopted an intensive daily diary design to investigate the double-edged effects of teachers’ anger. Multilevel structural equation modeling of data from 4,140 daily diary entries provided by 655 practicing Canadian teachers confirmed our hypotheses. Trait anger in teachers was found to impair teacher-perceived student engagement. Daily genuine expression of anger corresponded with greater teacher-perceived student engagement; daily faking anger impaired perceived student engagement, and daily hiding anger showed mixed results. Moreover, teachers tended to hide anger over time, and were reluctant to express anger, genuine or otherwise, in front of their students. Finally, genuine expression and hiding of anger had only a temporary positive association with teacher-perceived student engagement, with student rapport being optimal for promoting sustained observed student engagement.

## Introduction

Teachers experience a wide range of emotions in the classroom. However, whereas studies have consistently examined teachers’ experiences of positive emotions, such as enjoyment and enthusiasm (e.g., Frenzel et al., 2009, 2018; Keller et al., 2016; Taxer & Frenzel, 2018), there is relatively less attention on teachers’ negative emotions (e.g., Burić & Frenzel 2019). Among various types of negative emotions in teachers, existing research suggests that anger is the most frequently experienced (Frenzel, 2014; Keller et al., 2014b) and has mixed effects on student engagement. More specifically, habitual experiences of anger (*trait anger*) have been found to exhaust teachers’ cognitive resources and harm their emotional well-being (Chang, 2009), leading to poor instructional quality (Chang, 2009; Frenzel, 2014; Frenzel et al., 2021) and lower student engagement (Assor et al., 2005; Hagenauer et al., 2015; Klusmann et al., 2022). In contrast, teachers’ efforts to modulate how they express anger on a daily basis in the classroom (i.e., *emotional labor*) have been shown to promote teaching effectiveness as well as foster student concentration and engagement (e.g., Burić et al., 2019; Hagenauer & Volet, 2014; Sutton et al., 2009; Taxer & Gross, 2018).

To empirically examine the double-edged effects of teachers’ anger and corresponding emotional labor strategies (i.e., genuine expression, faking, hiding anger in class), we investigated their relative effects on perceived student engagement based on daily diary data from practicing teachers over a two-week period (ten consecutive teaching days). This research thus aims to contribute to a more comprehensive understanding of how teacher anger and the corresponding emotional labor can hinder or aid teaching and learning over time and across contexts (e.g., primary and secondary schools; diverse range of subjects).

## Theoretical perspectives on teacher anger

### The appraisal perspective

The cognitive appraisal theory of emotion (Lazarus, 1991; Lazarus & Folkman, 1984) has long served as a key conceptual framework underlying much of the existing research on teachers’ emotions in differentiating between two types of appraisals: primary and secondary (Frenzel, 2014; Frenzel et al., 2021). Primary appraisals determine the valence of emotional experiences (positive vs. negative). When teachers’ personal teaching goals and their appraisals of classroom events (i.e., students’ performance, motivation, discipline, and quality of teacher-student relationships) are in alignment, teachers are expected to experience more positive emotions. However, when their teaching goals and classroom realities are discordant, teachers are instead found to experience negative emotions (e.g., Becker et al., 2015; Frenzel et al., 2021).

In contrast, secondary appraisals represent teachers’ perceived potential to cope with adverse classroom events (e.g., a student disrupts a lesson) and their perceived ability to change negative classroom events to bring them more in line with their teaching goals (Berkowitz & Harmon-Jones, 2004). Teachers who view such events as uncontrollable often experience feelings of anxiety, fear, or frustration and are less likely to take action to solve the problem (*flight response*; Lerner & Keltner 2001; Wang & Hall, 2018). In contrast, teachers with greater perceived competence to address the situation tend to view it as more controllable, feel anger due to the unfairness of the event, and are more likely to attempt to solve the problem (*fight response*; Chang 2009; Lerner & Keltner, 2001; Sutton et al., 2009). Whereas primary appraisals determine emotion valence (e.g., positive vs. negative), secondary appraisals involving coping potential and perceived controllability are hypothesized to determine emotional intensity (Lazarus & Folkman, 1984).

### Teachers’ values about connecting with students

Appraisal theories further suggest that an individual’s personal values should be an especially critical predictor of their subsequent emotions and behaviors (e.g., control-value theory of achievement emotions; Pekrun 2006). For example, teachers’ values pertaining to developing meaningful relationships with students have consistently been found to correspond with teachers’ emotions and instruction. Teachers who believe that maintaining close relationships with their students is important tend to adopt more mastery-oriented teaching strategies (e.g., recognizing individual students’ learning progress) rather than performance-oriented teaching approaches (e.g., focusing on current student performance), and experience more enjoyment and less anger in the classroom (Wang et al., 2017). Students of teachers who value close relationships with their students tend to perceive greater teacher support, higher quality of instruction (e.g., more cognitively stimulating strategies), and are more likely than others to seek teachers’ help (Butler & Shibaz, 2014). As a result, these socially motivated teachers are more likely to perceive an alignment between their personal teaching goals and classroom events, leading to more positive emotions (enjoyment), fewer negative emotions (anger), greater job satisfaction, and a lower likelihood of wanting to quit the profession (Frenzel et al., 2021; Wang & Hall, 2019).

### Teachers’ emotions, instructional quality, and student engagement

With respect to teaching-related emotions, appraisal theories additionally propose that teachers’ emotions and students’ classroom behaviors have a recursive relationship. According to the reciprocal model of teacher emotions (Frenzel, 2014; Frenzel et al., 2021), teachers’ emotions result from cognitive appraisals regarding the success or failure of their teaching efforts. In turn, these emotions impact teachers’ use of cognitively and motivationally stimulating teaching approaches, thus indirectly affecting subsequent student engagement that is once again appraised by teachers in relation to their instructional goals and values (Frenzel et al., 2009, 2018). In addition, teachers’ emotions are assumed to impact student engagement through emotional transactions (e.g., emotional labor, contagion) through which students infer critical information about their teachers’ beliefs that, in turn, impact students’ own beliefs and actions (Côté et al., 2013; Frenzel et al., 2021; Van Kleef, 2009). For example, a teacher who expresses anger to implicitly convey dissatisfaction with a student’s academic failure or classroom disruption may subsequently observe the student modifying their behavior by investing more effort in studying or ceasing disruptive behaviors.

In addition, teachers’ emotional experiences can also indirectly correspond with student engagement through instructional quality (e.g., Frenzel et al., 2021; Kunter et al., 2013). Teachers who experience positive emotions tend to have better instructional quality characterized by student-focused approaches. In contrast, teachers who regularly experience negative emotions tend to exhibit less effective instructional strategies (i.e., teacher- vs. student-focused approaches) that, in turn, erode student engagement (Frenzel et al., 2021). Concerning teachers who regularly experience anger (*trait anger*), this emotion has been found to correspond with adverse physiological attributes (high blood pressure, body temperature, heart rate) and facial expressions in teachers as observed by students (furrowed eyebrows, clenched teeth, flushed cheeks; Harmon-Jones et al., 2016). Persistent feelings of anger have also been found to deplete cognitive resources in teachers, hindering their concentration and instructional decision-making (Beach & Pearson, 1998; Emmer, 1994; Eysenck & Calvo, 1992; Frenzel, 2014; Sutton & Wheatley, 2003).

Empirical findings further show higher trait anger in teachers to be associated with student-perceived lack of clarity in instruction, lower instructional content relevance (Becker et al., 2014), and a lack of variety in instructional strategies (Frenzel et al., 2016, 2021). Studies also show frequent experiences of anger to correspond with lower self-efficacy for teaching (Sutton et al., 2009), less enthusiasm for instruction (Wang & Hall, 2021), shallow information processing (Frenzel et al., 2021; Wilkowski et al., 2010), and poorer supervisor-rated teaching performance (among preservice teachers; Chen 2019). Not surprisingly, poor instructional quality is also closely linked with more maladaptive student outcomes. More specifically, teachers who evidence poorer instructional strategies tend to be less competent in managing challenging classroom behavior, provide less effective student support, and elicit poorer cognitive engagement in their students (Kunter et al., 2013), leading to deteriorated student progress (Blömeke et al., 2022), motivation (e.g., interest, self-concept; Klusmann et al., 2022), and academic achievement (König et al., 2021).

## Emotional labor of anger in teachers

Teachers’ experiences and regulation of anger represent distinct processes such that after experiencing feelings of anger, teachers may intentionally attempt to express or suppress it. Teachers may also attempt to fake an expression of anger with students as a strategic attempt to manage classroom misbehavior. These attempts by teachers to intentionally convey emotional expressions to students that differ from their truly experienced emotions are referred to as *emotional labor*, with such expressions often determined by emotional display rules specific to the teaching profession (Hochschild, 1983; Wang et al., 2019; Yin et al., 2019).

Traditionally, teachers’ emotional labor has been conceptualized as involving two main strategies: deep acting and surface acting. Deep acting concerns how individuals actively and purposefully modify their felt emotions so that the emotions experienced and expressed coincide. In contrast, surface acting refers to individuals faking unfelt emotions or suppressing felt emotions such that the displayed emotions are aligned with desired emotions (Brotheridge & Grandey, 2002; Brotheridge & Lee, 2003; Hochschild, 1983). However, this traditional dichotomous conceptualization of emotional labor is limited as it does not account for emotion valence (e.g., positive vs. negative emotions), specific emotion types (e.g., anger vs. anxiety), or specific types of emotion regulation strategies (e.g., hiding vs. faking; Hagenauer & Volet 2014; Taxer & Frenzel, 2015; Wang et al., 2020). Accordingly, recent studies on teachers’ emotional labor have started to explore the complexities of this construct by assessing how specific strategies are used to express specific emotions, such as why teachers choose to *hide* observable signs of anger in class or, alternatively, *fake* or *genuinely express* it for classroom management purposes (e.g., Taxer & Frenzel 2015; Wang et al., 2020).

### Promoting student engagement through teachers’ emotional labor of anger

Teachers often choose to suppress feelings of anger due to experiencing it as an adverse, unwanted emotion, with losing one’s temper in front of students considered particularly shameful (Sutton, 2007; Sutton et al., 2009). Research on emotion transmission in class further suggests that students regularly mimic their teachers’ emotional expressions and make social appraisals based on their teachers’ expressed emotions (e.g., lack of expressed anger suggests the instructor is satisfied with students’ work; Frenzel et al., 2021; Hatfield et al., 1994; Manstead & Fischer, 2001; Parkinson & Manstead, 2015). Studies also indicate that teachers may hide feelings of anger to help them stay focused on teaching and maintain a productive learning environment that fosters student engagement (instrumental goals; Hagenauer & Volet 2014; Sutton, 2004; Taxer & Gross, 2018).

Unlike other negative emotions that are associated with avoidance motivational tendencies (i.e., feelings of anxiety prompting a flight response; Carver & Harmon-Jones 2009), findings suggest that the emotion of anger can, in fact, be stimulating and accompanied by high levels of perceived coping potential or controllability (Pekrun, 2006; Rivers et al., 2007; Weiner, 2010). Teachers who experience anger can thus be expected to want to change the situation and adopt an active approach to solving the problem (Burić & Frenzel, 2020). This approach motivation tendency of anger suggests that individuals may also express this emotion to others to convey important information concerning their motivation and beliefs (Van Kleef, 2009). Therefore, teachers may choose to instrumentally express their anger to help them deal with the anger-eliciting situation, so as to imply to students that they attribute poor performance or disruptive behaviors as controllable and potentially improved through effort. Such expressions of teachers’ anger are, in turn, expected to influence their students’ own causal attributions for their performance (e.g., increase controllable attributions) and foster emotional and behavioral engagement in class (see Wang & Hall 2018 for a review on teachers’ attributions).

Unlike the genuine expression of anger, in which teachers internally experience substantial levels of anger, faking anger suggests that the underlying emotion is less present; that their internally experienced emotions may instead be neutral or even positive (Lennard et al., 2019). It also implies that the classroom situation in question might not actually be anger-eliciting for the teacher. For example, although students might have already performed or behaved acceptably, a teacher may nevertheless decide to convey some degree of anger to imply that expectations remain unmet and more effort is required.

However, although both genuine expression of anger and faking anger can signal to students that greater progress and effort are expected, they nevertheless represent two different emotional labor strategies, with the former being the true expression of an emotion and the latter being a subtype of surface acting (Grandey & Melloy, 2017; Taxer & Frenzel, 2015; Wang et al., 2020). Prior research further suggests that students may be able to accurately determine the authenticity of teachers’ emotions from their verbal expressions and gestures, and respond accordingly. Inauthentic emotions are generally less effective than authentically expressed emotions for engaging students, with inauthentic expressions of emotion also found to have adverse effects on student learning (Keller et al., 2018).

## Emotional labor as a daily practice of teaching

The vast majority of research examines emotional labor from a trait perspective, suggesting that teachers tend to express or regulate emotions consistently independent of the classroom context. However, generalized trait reports (e.g., self-report questionnaires) can be biased due to self-deception or recollection errors, resulting in discrepancies from state or in situ experiences ((Keller et al., 2014a). In addition, state and trait assessments of the same construct can lead to substantially different relations with outcome variables (Goetz et al., 2015; Ripski et al., 2011). For example, whereas expressions of anger (genuine expression or faking) assessed at the state (within-person) level may lead to short-term gains in student engagement (e.g., Hagenauer & Volet 2014), long-term expression of anger (assessed at the trait or between-person level) may lead to poorer student-teacher relationships and lower student ratings of instructional quality (Frenzel et al., 2016). Similarly, although temporary suppression of anger (assessed at the state or within-person level) can foster concentration in both students and teachers and help accomplish instructional goals (e.g., Hagenauer & Volet 2014), long-term suppression of anger assessed at the trait (between-person) level tends to correspond with lower student engagement (e.g., Burić et al., 2019). Persistently hiding unpleasant emotions thus not only consumes teachers’ cognitive resources and impairs instruction over time (Hülsheger & Schewe, 2011), but students are also more likely to detect and be negatively impacted by insincere displays of emotions from teachers over an extended period.

### State characteristics of teachers’ emotional labor

Recent conceptualizations of both emotion and emotional labor have emphasized the dynamic nature of these constructs that may fluctuate as a function of time or teaching context (Beal & Trougakos, 2013; Grandey & Gabriel, 2015; Gross, 2015; Kuppens & Verduyn, 2015). However, studies that assess variability in these constructs over time through experience sampling or daily diaries remain scarce (cf. Sonnentag & Starzyk, 2015). For example, Carson’s (2006) pioneering research using state assessments with practicing teachers (*N* = 44) found that momentary emotions and emotion regulation frequencies across occasions were related to teacher burnout. Similarly, an experience sampling study by Keller and colleagues (2014b) with German teachers (*N* = 39) found that teachers’ momentary experiences of enjoyment, anger, and anxiety predicted momentary emotional labor strategies (i.e., surface acting).

More recently, a diary study by Lavy and Eshet (2018) with Israeli teachers (*N* = 62) found that teachers’ daily experiences of positive emotions promoted adaptive emotion regulation, triggering upward emotional spirals and heightened well-being. Moreover, a recent study conducted by de Ruiter et al. (2021) found that Dutch teachers (*N* = 37) used various emotional labor strategies on a daily basis in response to specific instructional events (e.g., the valence of events) and their perceived relationships with students. Although these studies are limited by small sample sizes at the teacher level, such findings clearly support the assertion that teachers do not engage in the same emotional labor strategies over time but instead adjust their emotional expressions from day to day, or depending on the teaching context, to achieve their instructional goals.

There is currently very limited empirical research investigating the relationship between teachers’ state emotional labor of anger and perceived student engagement. Nevertheless, qualitative findings from interviews with teachers do suggest that teachers may selectively hide or express (genuine or not) anger in the classroom according to specific instructional situations and to achieve specific instructional goals. For example, Hagenauer and Volet’s (2014) interviews with Australian teachers showed that teachers choose to suppress versus express anger depending on the context to maintain classroom order. Taxer and Gross’ (2018) study with US teachers similarly showed that teachers may choose to fake anger in specific situations such as when other students are negatively impacted (e.g., a funny joke was made at another student’s expense) or for classroom management purposes (e.g., to prevent escalation of group misbehavior). As such, limited evidence suggests that whether it is faking, hiding, or genuine expression of anger, such strategies may be instrumentally and effectively used by teachers in specific instructional contexts to bring about positive changes in student engagement.

### Trait characteristics of teachers’ emotional labor

In addition to the state characteristics of teachers’ emotional labor, teachers’ emotional labor also shares trait characteristics. On the one hand, it is possible that a teacher may strategically choose the specific emotional labor strategy of conveying anger (e.g., faking, genuine expression) at a specific time to increase student engagement, such as in an underperforming class at the beginning of the week (state emotional labor). On the other hand, it is also possible that a teacher may consistently choose to hide feelings of anger when teaching at-risk students throughout an entire semester to prevent student disengagement (trait emotional labor). Thus, although teachers’ emotional labor strategies may *fluctuate* depending on the instructional context, it is reasonable to expect that teachers may nevertheless adopt *similar* emotional labor strategies under the same classroom situations, or with the same group of students, over an extended period of time.

The use of trait measures to assess teachers’ emotional labor implies that teachers regularly adopt similar emotional labor strategies over time and across contexts. For example, findings from Wang et al. (2021) showed practicing teachers to report consistent levels of genuine expression, faking, and hiding of emotions (positive and negative) over time based on trait measures assessed at two-time points (five-month lag). Similar results on trait measures were observed in longitudinal studies by Hülsheger et al. (2010) and Philipp and Schüpbach (2010) that found teachers to use surface and deep acting strategies consistently over two-month and one-year lags, respectively. Following from these findings, it is reasonable to anticipate that teachers who express anger in class on a given day would also be more likely to express anger on other days. Conversely, teachers who express lower levels of anger in front of students on a given day would similarly be expected to express low anger levels on other days. In other words, teachers who are more likely than others to use a particular emotional labor strategy to deal with anger on a particular day are expected to similarly use this strategy *across* the following days. Such an assumption thus incorporates both state and trait characteristics of teachers’ emotional labor of anger in suggesting that despite some fluctuation over time, teachers’ emotional labor strategies across teaching days should remain relatively stable.

## The present study

The present study had three primary aims. First, we examined the extent to which teachers who reported more strongly valuing relationships with their students felt less anger overall (trait), and further, if lower trait anger corresponded with greater teacher-perceived student engagement. Second, we explored if teachers’ daily emotional labor of genuine expression, faking, or hiding of anger facilitated daily teacher-perceived student engagement. Third, we tested whether teachers’ emotional labor demonstrated both trait and state characteristics, such that although strategies may fluctuate from day to day, they were nevertheless expected to remain largely consistent across days and classes. Following from prior studies showing teacher demographics, including gender, years of teaching, and grade level of instruction to significantly correspond with teachers’ emotions and emotional labor strategies (e.g., Burić & Frenzel 2019; Taxer & Frenzel, 2018), these variables were additionally assessed as covariates. Moreover, based on existing research demonstrating significant negative correlations between teachers’ trait enjoyment and trait anger (e.g., Becker et al., 2015; Wang & Hall, 2021), a trait measure of teacher enjoyment was included as a covariate to rule out greater anger simply reflecting lower teaching-related enjoyment.

### Hypothesis 1

Teachers who value better relationships with their students are expected to feel lower levels of anger. This hypothesis is consistent with the control-value theory which proposes that an individual’s values are an especially critical predictor of their subsequent emotions in achievement settings (Pekrun, 2006). The hypothesis is also consistent with previous findings suggesting that teachers who value their relationships with students are more likely to perceive an alignment between their personal teaching goals and classroom events, and hence experience more positive emotions and fewer negative emotions pertaining to their teaching (Frenzel et al., 2021; Wang & Hall, 2019).

### Hypothesis 2

Teachers who regularly experience anger in the classroom should perceive their students to be less engaged. This hypothesis is consistent with previous research suggesting that negative teacher emotions can have both direct (e.g., emotional contagion, inferential processes; Frenzel et al., 2009, 2018, 2021) and indirect detrimental influences (e.g., via instructional quality, stimulating styles Frenzel et al., 2021; Kunter et al., 2013) on their (perceived) student engagement.

### Hypothesis 3

Teachers who *hide* their anger in class on a given day should also observe their students be *more* engaged that day (3a). Teachers who strategically express *genuine* feelings of anger in class on a given day should similarly observe their students as being *more* engaged on that day (3b). Teachers who *fake* feelings of anger in class on a given day are also expected to observe their students be *more* engaged that day, but to a lesser extent than when genuine anger is expressed (3c). These sub-hypotheses were consistent with prior, primarily qualitative studies in which teachers reported using all three strategies (genuine expression, faking, hiding) to deal with anger and found each of them to be useful for improving classroom dynamics (e.g., Hagenauer & Volet 2014; Taxer & Gross, 2018). However, due to notably limited empirical evidence pertaining to these assertions, these hypotheses are primarily exploratory in nature.

### Hypothesis 4

Teachers who have used a specific emotional labor strategy (e.g., genuine expression, hiding, or faking of anger) on a given day will be more likely to use the same strategy in the following days. This hypothesis is inferred from prior trait-based assessments of emotional labor which suggest that teachers may regularly adopt similar emotional labor strategies over time and across contexts (e.g., Philipp & Schüpbach 2010; Wang et al., 2021). Those who use a particular strategy to deal with anger on a particular day are expected to use the same strategy across days.

## Method

### Participants and procedures

Practicing teachers were recruited in collaboration with 22 teacher associations across five Canadian provinces and one territory (Quebec, Ontario, British Columbia, New Brunswick, Newfoundland and Labrador, Yukon). Emails and newsletter announcements were sent to association members with a link to the study survey preceded by a consent page outlining the study purpose, risks, benefits, and confidentiality. The study questionnaires were similarly administered online and included demographic items as well as self-report measures of trait anger, the emotional labor of anger, value for teacher-student relationships, and perceived student engagement.

The present study involves two phases—a trait phase and a daily diary phase. Phase 1 was conducted at the start of the semester with 1,086 teachers who completed a survey assessing their values pertaining to relationships with students and trait anger. Two weeks later, the same teachers were invited to participate in Phase 2 that consisted of a set of diary surveys assessing their daily emotional labor of anger and daily perceived student disengagement for ten consecutive teaching days. The Phase 2 sample consisted of 655 teachers^1^ (114 male, 538 female, three undisclosed genders) who were primarily Caucasian (94%) and with an average of 15.3 years of teaching experience (*SD* = 7.6). Most Phase 2 participants held either a bachelor’s degree (43.4%) or a master’s degree (25.5%), with most teaching either primary school (42.4%) or secondary school students (44.1%; the rest reported teaching at both primary and secondary levels). Phase 2 participants completed a total of 4,140 diary surveys (mean diary surveys per teacher = 6.32). Teachers who continued to the second phase did not differ significantly from those who withdrew from the study in gender (*b* = 0.230, *SE* = 0.823, *p* = .384), years of teaching experience (*b* = − 0.341, *SE* = 0.838, *p* = .367), ethnicity (*b* = 0.019, *SE* = 1.134, *p* = .399), educational degree (*b* = 0.312, *SE* = 0.242, *p* = .174), grade of instruction (*b* = − 0.723, *SE* = 0.921, *p* = .293), trait anger (*b* = − 0.081, *SE* = 0.041, *p* = .996), or student relationship value (*b* = -0.036, *SE* = 0.044, *p* = .253).

### Measures

#### Teaching-related trait anger

The current study utilized the Teacher Emotions Scale (TES) developed by Frenzel et al. (2016) to assess the dispositional tendencies of teachers to experience anger in the classroom (four items; e.g., “I often have reasons to be angry while I teach”). The measure employs a four-point Likert scale (1 = *Strongly disagree* to 4 = *Strongly agree*; α = 0.788).

#### Teacher-student relationship values

A three-item, five-point measure developed by Cable and Edwards (2004) and validated by Wang and Hall (2019) with K-12 teachers was used to measure teachers’ perceptions as to the importance of developing meaningful relationships with students (α = 0.762; 1 = *Not important at all* to 5 = *Extremely important*). Sample items include “developing close ties with students” and “getting to know my students quite well.”

#### Daily emotional labor of anger

An adapted version of Glomb and Tews’ (2004) Discrete Emotions Emotional Labor Scale (DEELS) assessed teachers’ efforts to fake, hide, or express genuine anger in instructional contexts^2^. In the daily questionnaire, teachers were first asked whether they were teaching on that day, with teachers who were not teaching redirected to the final survey page and not provided any questions. Teachers who reported teaching on that day were asked the following emotional labor questions: “Thinking about your teaching experience *today*, how often did you try…” (1) “to *express* each of the following emotions if you *did feel* that way?” (*Genuinely expressed emotions*); (2) “to *express* each of the following emotions when you *did not feel* that way?” (*Faking emotions*); and (3) *“not to express* each of the following emotions when you *did feel* that way?” (*Hiding emotions*). A list of emotions was subsequently presented to teachers following each question (e.g., enjoyment, anger, anxiety), with the current analyses limited specifically to expressing anger in the classroom (1 = *Never*, 2 = *Rarely*, 3 = *Sometimes*, 4 = *Often*, 5 = *Always*). On each diary survey date after the first, teachers were encouraged to respond to the survey items in reference to the same class to which their answers pertained on the preceding days.

#### Daily perceived student engagement

Two items selected from a four-point measure of student engagement developed by Skinner et al. (2009) assessed teachers’ daily perceptions of their students’ behavioral engagement (“In my class today, my students tend to work as hard as they can”) and emotional engagement (“In my class today, my students seem to enjoy it”; 1 = *Not at all true*; 4 = *Very true*). The preamble for this scale asked teachers to reflect on how engaged their students were in class overall, albeit specifically *on that teaching day*.

### Data analysis

#### Rationale for analyses

Missing data were estimated with *Markov Chain Monte Carlo multiple imputations* (Peugh & Enders, 2004) with the following specifications: single-chain, EM Posterior mode initial estimates, Jeffreys priors, 500 imputations, 200 burn-in iterations, and 100 iterations. *Confirmatory factor analyses* (Jöreskog & Sörbom, 2018) were further conducted to analyze whether sets of survey questions reflected intended underlying constructs (e.g., teacher-student relationship value). To assess CFA fit, we used the comparative fit index (CFI), Tucker–Lewis index (TLI), root mean square error approximation (RMSEA), and standardized root mean square residual (SRMR; Hu & Bentler 1999) with two fit thresholds: good (CFI & TLI > 0.95; RMSEA < 0.06; SRMR < 0.08) and moderate (0.90 < CFI & TLI < 0.95; 0.06 < RMSEA < 0.10; 0.08 < SRMR < 0.10). All analyses were conducted with LISREL 10.1 (Jöreskog & Sörbom, 2018).

*Multilevel analysis* was conducted to account for lessons taught by the same teacher likely resembling one another more than those taught by different teachers (*nested data*; Hox et al., 2017), with *Q-statistics* testing all groups for serial correlation in adjacent lessons (Ljung & Box, 1979). If the serial correlation of the outcome (e.g., *genuinely express anger*) was significant, the lagged outcome variable in the previous lesson was added (*genuinely express anger [–1]*) as an explanatory variable to remove the serial correlation (Chiu & Lehmann-Willenbrock, 2016). To address multiple outcomes potentially having correlated residuals that underestimate standard errors, we employed a *multivariate outcome multilevel analysis* (Hox et al., 2017) and a *multilevel structural equation model* (Jöreskog & Sörbom, 2018).

As responses from the preceding days might influence current day responses, previous days (*time context*) were modeled with a *vector auto-regression* (VAR, Kennedy 2008). Given that separate, single-level tests of indirect mediation with nested data can inflate Type I error, we tested simultaneous multilevel mediation effects with a *multilevel M-test* (MacKinnon et al., 2004) and a *multilevel structural equation model* (Little et al., 2012). To further mitigate Type I error, a *two-stage linear step-up procedure* was adopted (Benjamini et al., 2006) with differences in effect sizes of explanatory variables tested using *Lagrange multiplier tests* (Bertsekas, 2014). Finally, we tested whether day of response as an explanatory variable would significantly correspond with teachers’ perceived student emotional engagement or behavioral engagement, as well as whether teachers’ perceived student engagement significantly changed over time.

### Explanatory model rationale

A streamlined version of statistical discourse analysis was employed to identify how teacher anger was related to their perceptions of students’ emotional and behavioral engagement (Chiu & Lehmann-Willenbrock, 2016). A multilevel variance components model (Hox et al., 2017) was conducted to test for significant differences in the outcome variables across lessons/days (level 1) and teachers (level 2).

1 $$Engag{e_{yij}} = {\beta _y} + {f_{yj}} + {e_{yij}}$$

For the **Engage** vector, outcome *y* (*perceived emotional engagement of students* or *perceived behavioral engagement of students*) on day *i* by teacher *j* had an overall mean β_y_ and unexplained components (*residuals*) at the lesson and teacher levels (*f*_yj_, *e*_yij_).

2 $$\begin{array}{l}Engag{e_{yij}} = {\beta _y} + {f_{yj}} + {e_{yij}} + {\beta _{yu}}Demographic{s_{yj}} + {\beta _{yv}}Course{s_{yj}} + {\beta _{yw}}Teachin{g_{yj}}\\+ {\beta _{yxj}}Current\_lesso{n_{yij}} + {\gamma _{y\left( {i - 1} \right)j}}Earlier\_lesso{n_{y\left( {i - 1} \right)j}}\\+ {\phi _{y\left( {i - 2} \right)j}}Earlier\_lesso{n_{y\left( {i - 2} \right)j}} \ldots \end{array}$$

Vectors of structural explanatory variables were also assessed. We first entered **Demographics** variables including *female* (vs. male) and *years of teaching*. Next, we entered **Courses**, assessed as *secondary school and mixed grades* (baseline = primary), followed by relatively stable **Teaching** variables: *teacher-student relationship value, trait enjoyment*, and *trait anger*. We subsequently added short-term, emotional labor variables pertaining to the **Current_lesson**: *genuinely express anger, hide anger*, and *fake anger*. As teachers’ emotional labor in the preceding days are likely to impact teachers’ observation of their student engagement on the current day, we included these variables in reverse chronological order: **Earlier_lesson**_y(i − 1)j_, **Earlier_lesson**_y(i − 2)j,_ …. We tested each progressively earlier lesson until no variables in a lesson were significant (i.e., no variables from four days prior were significant).

We used multilevel mediation tests to create a multilevel path analysis (Hox et al., 2017) that served as an initial candidate for the ML-SEM (Jöreskog & Sörbom, 2018). The *total effect* (TE) of an explanatory variable on the outcome represented the sum of its direct effects (DE) and all indirect effects (IE). The indirect effect of explanatory variable X on outcome Y via mediator M [X → M →Y] was assessed as the product of the standardized parameter linking X to M multiplied by the total effect of M on Y. Non-significant demographic and teaching variables were omitted in the final model to reduce multicollinearity (Kennedy, 2008), and residuals were examined for significant outliers.

## Results

### Factor analysis and correlations

As the school level of each teacher variable showed no significant variance, an ML-SEM was not required, and a single-level CFA was conducted. All factors showed a good-moderate fit with high-reliability coefficients (all *R*_*c*_ exceeded 0.86; see Table 1), high factor loadings (mean = 0.80, minimum = 0.62), as well as small standard errors and uniqueness (see Table 2). Correlations, variances, and covariances between study variables are presented in Table 3. Teachers’ perceived student emotional and behavioral engagement showed a strong positive correlation (*r* = .786), with trait assessments of teachers’ enjoyment and anger having a strong negative correlation (*r* = − .551). The three emotional labor strategies for anger were moderately intercorrelated (*r*s = 0.291-0.360; Cohen 1988). Descriptive statistics for the survey variables are outlined in Table 4 and fit indices for latent analyses of trait measures are presented in Table 1.

**Table 1:** Goodness of Fit Measures for Congeneric Confirmatory Factor Analysis

Teachers’ trait factors	#	*R*_*c*_	α	SRMR	CFI	IFI	TLI	RMSEA	*χ* ^*2*^	*df*	*p*	AGFI	RFI
Anger	4	0.867	0.788	0.015	0.999	0.999	0.997	0.022	2.6	2	0.270	0.996	0.988
Enjoyment^+^	4	0.887	0.762	0.014	1.000	1.000	1.000	0.005	2.0	2	0.362	0.997	0.992
Student relationship value	3	0.918	0.846	0.014	1.000	1.000	0.998	0.024	2.7	2	0.256	0.997	0.994

***Note.***^+^Covariate; # = number of variables; R_c_ = reliability coefficient; α = Cronbach’s alpha; SRMR = standardized root mean square residual; CFI = comparative fit index; IFI = incremental fit index; TLI = Tucker–Lewis index; RMSEA = root mean square error approximation; df = degrees of freedom; AGFI = adjusted goodness of fit index; RFI = relative fit index. Each set of variables for a factor was modeled separately (congeneric)

**Table 2:** Factor Loadings, Standard Errors, and Uniqueness for Study Variables

Trait Variable	Standardized factor loadings	SE	Uniqueness
Trait Anger			
I often have reasons to be angry while I teach	0.842	0.027	0.291
I often feel annoyed while teaching	0.818	0.027	0.330
Sometimes I get really mad while I teach	0.718	0.034	0.485
Teaching generally frustrates me	0.706	0.038	0.502
Trait Enjoyment^+^			
I generally enjoy teaching	0.900	0.029	0.190
I generally have so much fun teaching that I gladly prepare and teach my lessons	0.807	0.029	0.349
I generally teach with enthusiasm	0.625	0.046	0.610
I often have reasons to be happy while I teach	0.726	0.034	0.473
Student Relationship Value			
Forming relationships with students	0.827	0.027	0.315
Getting to know my students quite well	0.899	0.021	0.191
Developing close ties with students	0.910	0.022	0.172

***Note.***^+^Trait enjoyment is analyzed as a covariate in the current study

**Table 3:** Correlations, Variances, and Covariances of Key Variables in the Lower Left, Diagonal, and Upper Right Matrices (N = 4,140)

	1	2	3	4	5	6	7	8	9	10	11	12	13
Student engagement													
1. Emotional engagement	**0.929**	0.723	-0.012	0.356	0.048	-0.040	-0.009	0.033	0.060	-0.053	0.103	0.238	0.005
2. Behavioral engagement	0.786	**0.911**	-0.011	0.459	0.027	-0.011	-0.017	0.032	0.073	-0.089	0.057	0.164	-0.009
Teachers													
3. Female teacher	-0.033	-0.030	**0.144**	-0.083	-0.034	0.030	0.005	-0.042	-0.014	0.009	-0.008	-0.002	0.012
4. Years of teaching	0.049	0.064	-0.029	**56.853**	-0.066	0.187	-0.120	-0.096	0.175	-0.448	-0.512	-0.334	0.032
5. Teach primary	0.101	0.058	-0.182	-0.018	**0.246**	-0.199	-0.046	0.095	0.006	0.008	0.043	0.007	0.009
6. Teach secondary	-0.082	-0.022	0.157	0.050	-0.807	**0.248**	-0.049	-0.081	0.012	-0.023	-0.048	-0.020	-0.008
7. Teach multiple grades	-0.030	-0.057	0.039	-0.052	-0.303	-0.318	**0.095**	-0.013	-0.018	0.014	0.005	0.014	0.000
8. Student relationship value	0.047	0.045	-0.150	-0.018	0.262	-0.223	-0.060	**0.530**	0.075	-0.052	-0.006	0.005	-0.004
Trait emotions													
9. Enjoyment	0.123	0.150	-0.072	0.045	0.022	0.048	-0.112	0.200	**0.262**	-0.177	-0.014	-0.105	0.002
10. Anger	-0.088	-0.149	0.036	-0.095	0.026	-0.073	0.075	-0.115	-0.553	**0.391**	0.091	0.125	0.001
Emotional labor strategies													
11. Genuinely express anger	0.103	0.057	-0.020	-0.066	0.085	-0.094	0.015	-0.008	-0.026	0.140	**1.071**	0.386	0.290
12. Hide anger	0.198	0.138	-0.003	-0.035	0.011	-0.033	0.036	0.006	-0.163	0.159	0.298	**1.562**	0.283
13. Fake anger	0.007	-0.011	0.039	0.005	0.022	-0.022	-0.001	-0.007	0.004	0.003	0.360	0.291	**0.607**

**Table 4:** Summary Statistics

Variable	*M*	*SD*	Min	Median	Max	*ICC*	Skewness	Kurtosis
Daily student engagement								
Emotional engagement	3.322	0.616	1.000	3.000	4.000	34%	− 0.512	0.193
Behavioral engagement	3.125	0.705	1.000	3.000	4.000	33%	− 0.508	0.271
Trait measures								
Student relationship value	4.267	0.740	1.000	4.372	5.000	-	-1.177	1.572
Enjoyment	3.408	0.519	1.214	3.600	4.000	-	− 0.709	0.153
Anger	1.665	0.632	1.000	1.521	4.000	-	1.059	0.716
Daily emotional labor								
Genuinely express anger	2.952	1.816	1.000	2.000	6.000	43%	0.687	− 0.936
Hide anger	3.283	1.720	1.000	3.000	6.000	41%	0.309	-1.098
Fake anger	2.270	1.944	1.000	1.000	6.000	42%	1.238	− 0.210

***Note***. *ICC* = intraclass correlation. Analyses included 655 teachers and their 4,140 daily responses

### Explanatory model

Most of the outcome variance occurred across days (student emotional engagement: 66%; student behavioral engagement: 67%) rather than across teachers (student emotional engagement: 34%; student behavioral engagement: 33%). Moreover, day of response was not found to significantly correspond with teachers’ perceived student engagement, and perceived student engagement did not significantly change over time: *r*(time, emotional engagement) = 0.01; *r*(time, behavioural engagement) = 0.09). As the lagged variables (e.g., hide anger [-1, -2, -3]) were highly correlated, the two-level SEM did not converge to a solution. However, the single-level SEM showed a good fit (SRMR = 0.059; CFI = 0.952; TLI = 0.942; RMSEA = 0.047; χ^2^[225] = 2,340; *p* < .001; IFI = 0.952; AGFI = 0.942; RFI = 0.936; see Fig. 1, panels a and b; see details in the supplementary file).

**Fig. 1:**
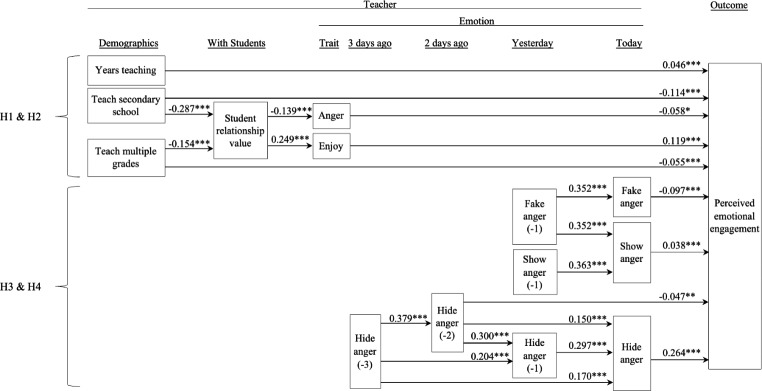
Panel a *Teacher Anger and Student Emotional Engagement* *p < .05, **p < .01, ***p < .001. H1-H4: Hypothesis 1-Hypothesis 4

**Fig. 1:**
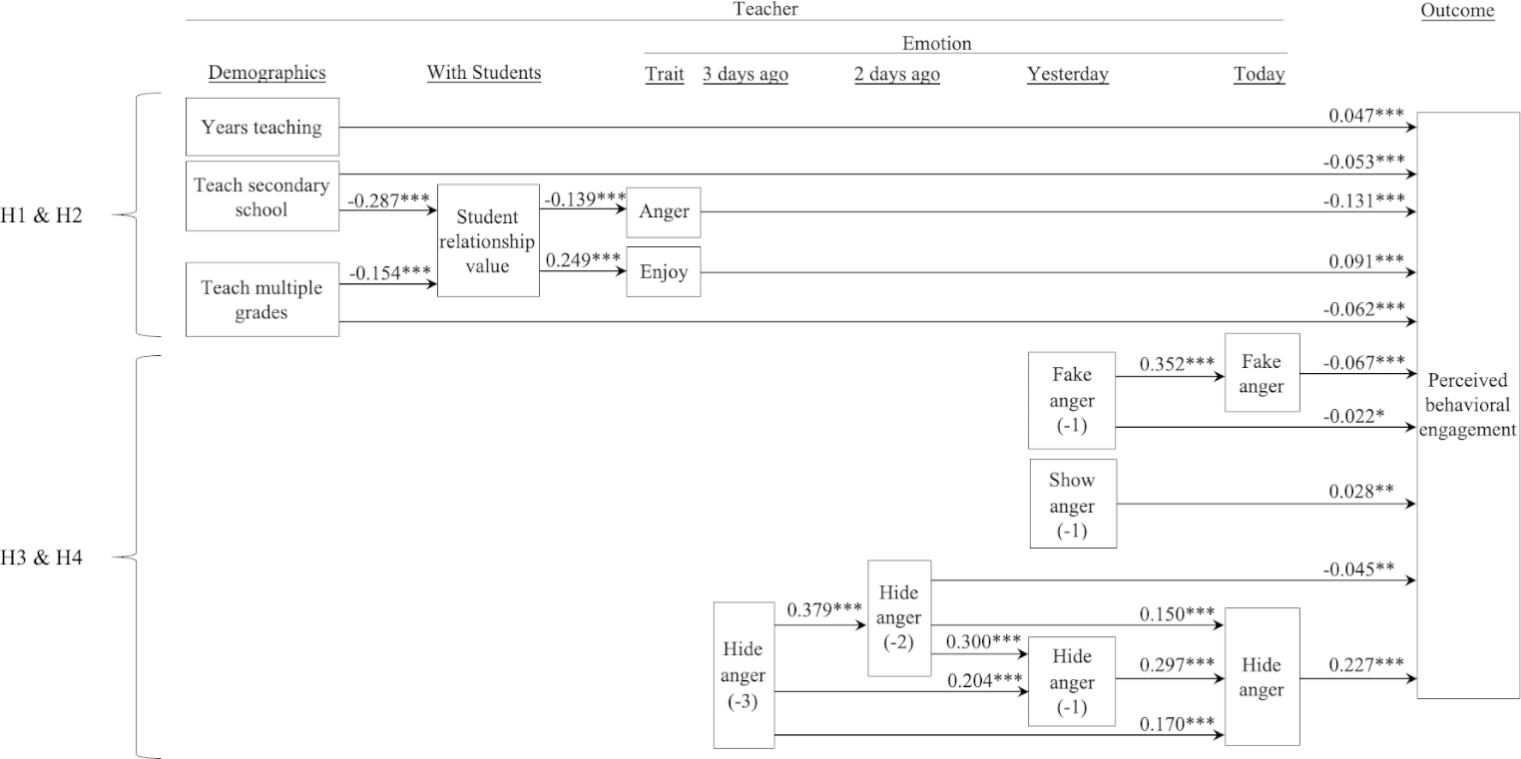
Panel b *Teacher Anger and Student Behavioral Engagement* ^**+**^Covariates; *p < .05, **p < .01, ***p < .001. H1-H4: Hypothesis 1-Hypothesis 4

#### Demographics and perceived student engagement

We present our results concerning teacher emotions and emotional labor of anger, as well as their relationships with teachers’ observation of student behavioral and emotional engagement (see Fig. 1). We present the results of our single, explanatory model of both types of perceived engagement in separate panels for ease of interpretation. See direct, indirect, and total effects in Table 5. For the purposes of clarity and parsimony, this table only shows significant paths. For details regarding the standardized and unstandardized parameters, and standard errors, see the supplementary file.

**Table 5:** Direct, Indirect, and Total Effects of Teacher Explanatory Variables on Student Engagement

	Student outcomes						
	Emotional engagement				Behavioral engagement		
Teacher explanatory variable/factor	Direct	Indirect	Total		Direct	Indirect	Total
Demographics							
Years teaching a^+^	0.046		0.046		0.047		0.047
Teach secondary school^+^	-0.114	-0.011	-0.125		-0.053	-0.012	-0.065
Teach multiple grades^+^	-0.055	-0.006	-0.060		-0.062	-0.006	-0.069
Student relationship value		0.039	0.039			0.041	0.041
Trait emotions							
Enjoyment^+^	0.123		0.123		0.091		0.091
Anger^a^	-0.058		-0.058		-0.131		-0.131
Emotional labor							
Genuinely express anger	0.038		0.038				
Hide anger	0.264		0.264		0.227		0.227
Fake anger	-0.097		-0.097		-0.067		-0.067
Genuinely express anger (-1)		0.014	0.014		0.028		0.028
Fake anger (-1)		-0.034	-0.034		-0.022	-0.024	-0.045
Hide anger (-1)		0.078	0.078			0.067	0.067
Hide anger (-2)	-0.047	0.063	0.016		-0.045	0.054	0.009
Hide anger (-3)		0.067	0.067			0.056	0.056

***Note.***^a^Blank cells indicate no significant effect. ^+^Covariates. Only significant findings are presented

The results suggest that teachers with more years of teaching experience and teachers of younger students reported greater student engagement. Years of teaching experience had a total, direct effect on both student emotional engagement (both TE [total effect] and DE [direct effect] = 0.046) and behavioral engagement (DE = 0.047; Table 5, top; Fig. 1, panels a and b, top; see details in the supplementary file). Compared to teachers of primary students, teachers of secondary students reported observing lower engagement. These effects were largest for teachers at secondary school students for both emotional engagement (TE = -0.125; DE = -0.114; IE [indirect effect] = -0.011 via lower teacher value for student relationships) and behavioral engagement (TE = -0.065; DE = -0.053; IE = -0.012 via lower teacher value for student relationships; Fig. 1, panels a and b, top left). For teachers who taught both primary and secondary school students, their students’ emotional engagement was between engagement levels observed by primary and secondary school students (TE = -0.060; DE = -0.055; IE = -0.006 via lower enjoyment; Fig. 1, panel a, top middle), with similar negative results found for behavioral engagement (TE = DE = -0.062; Fig. 1, panel b, top middle).

#### Hypotheses 1 and 2: teacher-student relationships, trait emotions, and perceived student engagement

Teachers’ values pertaining to developing meaningful relationships with students were linked to their trait emotions (enjoyment and anger) that, in turn, corresponded with their observed student engagement. Specifically, teachers who reported more strongly valuing relationships with students perceived their students to be more emotionally engaged (IE via greater teacher trait enjoyment [0.030] and lower trait anger [0.008]) and more behaviorally engaged (IE via greater trait enjoyment [0.023] and lower trait anger [0.018]; Fig. 1, panels a and b, top), supporting H1. Teachers who experienced greater enjoyment also had students who showed more emotional engagement (TE = DE = 0.123) and behavioral engagement (TE = DE = 0.091; Fig. 1, panels a and b, top middle). Conversely, teachers who experienced more trait anger reported students showing less emotional engagement (TE = DE = -0.058) and behavioral engagement (TE = DE = -0.131; H2; Fig. 1, panels a and b, top middle).

#### Hypothesis 3: emotional labor of anger and teacher-perceived student engagement

During the lesson, teachers’ emotional labor affected their observations of student engagement. When teachers genuinely expressed anger, their students showed slightly greater emotional engagement (TE = DE = 0.038; H3b; Fig. 1, panel a, right middle). When teachers hid their anger, their students showed much greater emotional engagement (TE = DE = 0.264; H3a; Fig. 1, panel a, right bottom) and behavioral engagement (TE = DE = 0.227; H3a; Fig. 1, panel b, right bottom). When teachers faked anger, their students showed slightly less emotional engagement (TE = DE = -0.097) and behavioral engagement (TE = DE = -0.067; Fig. 1, panels a and b, right middle; failing to support H3c). Hiding anger showed the largest effect on teachers’ perceived emotional engagement in their students (see Table 5 and supplementary file).

#### Hypothesis 4: daily practice of emotional labor

***The previous day.*** Teachers’ emotional labor, as reported on a given school day, was linked to perceived student engagement one day later. If a teacher showed anger on a given day (day − 1), that teacher was more likely to show anger the following day (day 0; H4) and, in turn, observe slightly higher emotional engagement (TE = IE = 0.014) and behavioral engagement levels that next day (day 0; TE = IE = 0.028; Fig. 1, panels a and b, right middle). If a teacher faked anger on a given day (day − 1), that teacher was also more likely to fake anger the next day (day 0; H4) and, in turn, observe slightly lower emotional engagement (TE = IE = -0.034) and behavioral engagement that next day (day 0; TE = -0.045; DE = − 0.022; IE = -0.024; Table 5, bottom). Finally, when a teacher reported hiding anger on a given day (day − 1), that teacher was more likely to hide anger the next day (day 0; H4) and, in turn, perceive slightly higher emotional engagement (TE = IE = 0.078) and behavioral engagement in their students that next day (day 0; TE = IE = 0.067; Table 5, bottom).

***The previous two days.*** Teachers’ emotional labor was also linked to teachers’ observations of student engagement two days later (Fig. 1, panels a and b, bottom, middle). Specifically, a teacher who reported hiding their anger on a given day (day − 2) was more likely to hide their anger both one day later (day − 1) and two days later (day 0; H4), leading to slightly higher perceived student emotional engagement (TE = 0.016; DE = − 0.047; IE = 0.063) and behavioral engagement two days later (day 0; TE = 0.009; DE = − 0.045; IE = 0.054; Table 5, bottom).

***The previous three days.*** Teachers who hid their anger on a given day (day − 3) were more likely to hide anger both the next day (day − 2), two days later (day − 1), and three days later (day 0; H4), resulting in students being observed by teachers to exhibit slightly higher emotional engagement (TE = IE = 0.067) and slightly higher behavioral engagement three days later (day 0; TE = IE = 0.056; see Fig. 1, panels a and b, bottom, middle).

This SEM accounted for 11% of the variance (squared multiple correlations) in student emotional engagement and 10% of the variance in student behavioral engagement. All other variables and factors were not significant. For example, neither prior day’s emotional engagement (-1) nor behavioral engagement (-1) was significantly linked to current day’s emotional engagement or behavioral engagement. Likewise, all other indirect mediation effects were not significant. Finally, the examination of residuals showed no substantial outliers.^3^

## Discussion

### Teacher-student relationships and teacher trait anger

The current study showed that teachers who value maintaining close relationships with their students tended to experience lower anger, thus supporting Hypothesis 1. These results are consistent with theoretical frameworks suggesting individuals’ value appraisals to lead directly to distinct emotional experiences (e.g., control-value theory of achievement emotions; Pekrun 2006). Prior empirical studies similarly show that teachers who more strongly value developing meaningful relationships with their students not only report greater job satisfaction and career commitment (Wang & Hall, 2019; Watt & Richardson, 2007, 2008), but also adopt more effective instructional strategies (i.e., mastery-oriented approaches), and report greater student engagement (Chang et al., 2022; Wang et al., 2017). As such, our findings expand upon existing research in further suggesting that teachers’ values pertaining to quality teacher-student relationships may also impact strong negative emotions in the classroom, such as anger. It is therefore possible that valuing close relationships with students may also lead to improved actual relationships with students and instructional effectiveness, yielding greater student engagement and positive emotions and, in turn, greater enjoyment and lower anger in teachers.

### Teacher anger as a double-edged sword

#### Harmful effects of trait anger in teachers

Findings from the current study suggest that trait anger in teachers directly corresponds with lower perceived student engagement levels (emotional and behavioral) thus directly supporting Hypothesis 2. Such findings are consistent with theories that propose trait anger to exhaust cognitive resources and impair effective information processing (e.g., Wilkowski et al., 2010). Accordingly, our findings support this assertion in demonstrating that teachers who are overly occupied by anger are less likely to engage with their students effectively. Moreover, the current findings are also consistent with Frenzel et al.’s (2021) theoretical premises which suggest that teacher emotions can influence student outcomes (e.g., motivation, discipline, engagement) through direct transmission processes or indirectly through teachers’ instructional behaviors. The findings thus supported and extended this theoretical framework by showing teacher trait enjoyment (covariate) to directly correspond with greater teacher-perceived student engagement and teacher trait anger to correspond with poorer perceived student engagement. It is also important to note that although the current study examined whether trait anger impairs perceived student engagement, the alternative path may also occur such that perceiving students as disengaged may also trigger teacher anger.

#### Benefits of teachers expressing genuine anger

When using emotional labor as an instructional strategy, teachers may intentionally choose to display anger (genuine or otherwise) or suppress their anger to achieve an instructional goal. Our results provide clear support for the benefits of teachers choosing to express genuine anger in class as outlined in Hypothesis 3b in showing this strategy to foster student engagement levels. More specifically, expressing anger in a given class was associated with higher levels of observed student engagement on not only that teaching day but also the following teaching day. As anger is associated with strong action tendencies and appraising the teaching situation as potentially controllable in nature, the expression of genuine anger to students transmits important information about the teachers’ belief that students’ behavior can and should improve, stimulating their students to act accordingly (Rivers et al., 2007). Such findings are consistent with extant empirical work on teacher attributions (Weiner, 2000; see Wang & Hall 2018 for a review on teacher attributions), suggesting that teachers’ expression of anger implies that they attribute students’ failure or disruptive behaviors as controllable and thus can potentially be improved through effort. The expressions of teachers’ anger transmit messages that influence students’ own causal attributions for their performance and behaviors (e.g., increase controllable attributions), fostering their emotional and behavioral engagement in class (Bibou-Nakou et al., 2000; Georgiou et al., 2002; Graham & Williams, 2009).

#### Mixed effects of teachers hiding anger

Compared to the expression of genuine anger, hiding anger was found to have stronger albeit mixed relations with daily student emotional and behavioral engagement, thus only partially supporting Hypothesis 3a. Teachers who hid their anger on a given day were more likely to perceive better emotional and behavioral engagement in their students on that day, with this strategy showing the strongest effects among three emotional labor strategies. However, teachers who reported hiding anger were also found to report slightly poorer student engagement two days later, suggesting that hiding anger may have temporary benefits but be detrimental in the long-term to the extent of engagement they see in their students (contradicting Hypothesis 3a).

These same-day results support prior research in suggesting that temporary suppression of anger may facilitate teacher-perceived student engagement. Teachers have often reported that hiding their anger and not venting during class can help them achieve instructional goals and facilitate student concentration (Hagenauer & Volet, 2014; Sutton et al., 2009; Taxer & Gross, 2018). However, most prior studies suggesting positive effects of teachers’ hiding emotions are primarily smaller-scale and qualitative in nature, with larger-scale, empirical replication studies in this domain having yet to be conducted. Our findings thus contribute to existing research on teachers’ efforts to hide emotions from students by providing a quantitative perspective showing that although hiding emotions may sometimes be necessary and effective, it is unlikely to represent a longer-term solution for engaging students in class and may instead have opposite results. Accordingly, these findings also extend upon prior research showing hiding emotions to be primarily maladaptive for teachers (see Yin et al., 2019 and Wang et al., 2019 for meta-analytic reviews) and students (e.g., Burić et al., 2019) in suggesting that hiding anger may indeed provide immediate student benefits in a given classroom context (e.g., when students appear particularly disengaged) despite long-term adverse effects on teachers and students.

#### Harmful effects of teachers faking anger

Our findings also suggest that faking anger was not an effective strategy for teachers for improving the level of engagement they observed in their students, with this strategy being associated with poorer emotional and behavioral engagement in students (thus failing to support Hypothesis 3c). Faking anger implies that the underlying emotional experiences are less negative, or perhaps even neutral or positive (Lennard et al., 2019). Accordingly, it is possible that the intentional display of upregulated anger may not prompt behavioral changes in students if they detect that the anger displayed is not sincere (e.g., Frenzel et al., 2021; Keller et al., 2018). Such findings also underscore the importance of emotional authenticity in interpersonal interactions between teachers and students (Frenzel et al., 2021). For example, Keller et al.’s (2018) study has found that students could indeed sense it when their teachers’ enjoyment and enthusiasm were faked or inauthentic, with such detection of teachers’ emotional inauthenticity associated with unfavorable student outcomes (e.g., less experience of enjoyment and more boredom). Moreover, another study by Taxer and Frenzel (2018) has found that pretending to be enthusiastic in the classroom was associated with poor teacher motivation (e.g., self-efficacy), adverse emotional experiences (e.g., more anger and anxiety), and poor occupational well-being (e.g., low job satisfaction and high emotional exhaustion).

In sum, the present results convey clear information that (1) genuine expression of anger can improve the level of engagement observed in students, (2) hiding anger has more mixed results (temporary benefit and long-term harm), and (3) faking anger is not an effective strategy for observing improvements in student engagement. Such findings thus support the overall study premise that teachers’ anger is a double-edged sword when examining its effects on students’ classroom engagement. Whereas habitual experiences of anger were maladaptive for improving perceived student engagement, instrumental and intermittent displays or hiding of anger had positive effects on teacher perceptions of student engagement. However, it is essential to note that these benefits of expressing or hiding of anger in class were short-lived, becoming non-significant after just one to two days. Accordingly, these findings suggest that teachers should consider other strategies for achieving sustained engagement levels of their students over time (e.g., supporting autonomy, mastery-oriented instruction, and emotional support; Assor et al., 2005; Klusmann et al., 2022; Schiefele, 2017).

### Emotional labor as a daily teaching practice

To our knowledge, the present study is the first to examine the daily patterns of teachers’ emotional labor on a large scale. Results suggest that teachers indeed tend to adopt similar strategies across days such that teachers who adopted one strategy on a given day were also more likely to use the same strategy up to three days later (e.g., hiding anger). These findings thus support Hypothesis 4 in showing that although teachers’ emotional labor has state characteristics and may fluctuate based on the daily instructional context, it is also trait-like, with the same strategies tending to be adopted similarly over time. This trait-like pattern was especially evident for teachers’ efforts to hide feelings of anger and was less prominent for genuine expression of anger and faking anger. A possible explanation for these results is that teachers often see anger as an inappropriate emotion in the classroom and regard losing their temper in front of students as shameful and unprofessional (Sutton et al., 2009). Therefore, it is perhaps not unexpected that teachers tend to consistently report hiding anger in class, and although they do acknowledge strategically expressing genuine anger, such expressions are found to be mostly temporary.

### Study limitations

Findings from the current study should be considered in light of limitations concerning study methods and design. First, as teachers’ value for student relationships, their levels of anger, and their perceptions of student engagement are often reciprocally related, it is empirically and practically challenging to determine cause-and-effect relationships (Frenzel et al., 2021). In the current study, we specifically analyzed how teachers’ values pertaining to teacher-student relationships are associated with feelings of anger that, in turn, predicted observed student engagement. However, reverse causal paths are also plausible. For example, perceived student engagement may predict teachers’ subsequent emotions, such as enjoyment or pride, and teachers’ emotional labor of anger may predict their future relationships with students. Accordingly, future studies administering daily assessments of not only emotional labor and perceived student engagement but also discrete emotional experiences (e.g., anger, enjoyment) and perceived teacher-student relationship quality are needed to better address the expected reciprocal relationships between these constructs.

Second, as the current diary study design relied exclusively on teachers’ self-reports, it is possible that common method variance and hindsight or social desirability bias may have impacted study findings (e.g., teachers may have reported notably high levels of perceived student engagement to not appear ineffective). Although such issues with self-report measures were mitigated by the present study adopting an intensive daily diary design that examined nonintrusive measures of emotional labor and perceived student engagement over a ten-day period, our key measures of teacher-student relationship values and feelings of anger were assessed in a trait-like manner in a single study phase. As such, future studies utilizing more objective observational methods are warranted to examine not only students’ engagement in class but also teachers’ emotions and emotional labor strategies (e.g., via third-party reports, classroom recordings) to provide multiple sources of data to triangulate study findings and increase robustness. It is also recommended for further studies to more intensively examine the moderating roles of both student characteristics (e.g., socioeconomic status) and school demographics (e.g., locations, size, ethnic diversity) to better ascertain the generalizability of the present study findings.

Third, although our study examined teachers’ expressing, faking, and hiding anger at the daily level, it did not assess how such strategies varied in response to more specific instructional contexts. For example, Frenzel et al. (2021) proposed that whereas teachers’ efforts to strategically express anger might be effective in feedback contexts (e.g., prompting students to work harder after poor performance), it may instead be less effective in classroom management contexts (e.g., in response to typical student disruptions) than other approaches (e.g., hiding anger). Considering the present results showing that expressing or hiding anger may be effective in engaging students, future studies are needed to examine the specific types of instructional situations under which these strategies would be most beneficial for students.

Finally, whereas the current study examined only student-focused outcomes, it is important to note that teachers’ emotional labor strategies may have noteworthy effects on teachers themselves. For example, although displays of inauthentic emotions, such as hiding anger or faking enthusiasm, might improve the extent to which student engagement is observed by teachers (e.g., Burić et al., 2019), such strategies are also expected to consume teachers’ cognitive resources and potentially contribute to emotional exhaustion in teachers (Hülsheger & Schewe, 2011; Wang et al., 2019; Yin et al., 2019). Future studies further investigating the impact of teachers’ emotional labor on teacher well-being and persistence is therefore critical to determine if their emotional labor of anger may also have ambivalent effects in contributing to observed student engagement while at the same time compromising their motivation and psychological health.

### Practical study implications

Our initial demographic findings showed that primary school teachers tended to most strongly value meaningful relationships with their students, with reported value for teacher-student relationships declining as the grade level of instruction increased. As a fundamental psychological need, interpersonal relationships play a critical role in students’ social-emotional development (Deci & Ryan, 2000). Therefore, the current study findings suggest that teachers of higher grades should be encouraged through orientations and professional development initiatives to maintain a focus on developing strong emotional connections with their students through emotional and psychological support. This assertion is supported by value congruence research showing teachers who feel supported by their administrators in valuing meaningful relationships with students to report greater job satisfaction as well as lower levels of exhaustion and quitting intentions (Wang & Hall, 2019). Given the present findings showing more experienced teachers to observe greater engagement in their students as compared to novice teachers, it is possible that mentorship programs in which experienced educators counsel new teachers may also be especially effective in helping less experienced teachers understand the importance of managing and expressing their anger effectively to better engage students (Callahan, 2016; Kajs, 2002; Villar & Strong, 2007).

Our findings also clearly demonstrate that teaching-related emotions are complex. Whereas researchers have often classified teachers’ emotions as either “good” or “bad” for students, mixed effects have been found for both positive and negative emotions. For example, the *too-much-of-a-good-thing effect* suggests that positive emotions enhance well-being and performance to a certain extent, after which the benefits deteriorate, or harmful effects are observed (Grant & Schwartz, 2011). Likewise, although moderate levels of negative emotions can facilitate concentration and improve performance (e.g., anxiety; Cassady & Finch 2020), more intense negative emotional experiences instead impair performance (Cheng & McCarthy, 2018). This study suggests that teachers’ anger similarly represents a nuanced and complex emotional experience, with dispositional anger typically harming teaching effectiveness, in contrast to instrumental and intermittent expressions of anger that can instead improve teaching performance. Such improvements in teaching performance may also lead to an upward spiral resulting in greater positive emotions, decreased negative emotions (e.g., anger), and better emotional well-being for students and teachers alike.

To summarize the study findings, as opposed to suggesting that teachers avoid anger entirely, our results suggest that teachers instead be informed of strategies for dealing with it more effectively in the classroom. More specifically, when intense anger is experienced and expressing it is deemed not appropriate, teachers can be encouraged to suppress it temporarily. However, as long-term suppression of anger harms well-being (Wang et al., 2019) and impairs performance (Burić et al., 2019), other adaptive emotion regulation strategies should be considered, such as deep acting (e.g., Grandey & Melloy 2017), cognitive reappraisal (Gross, 1998), or problem-focused coping strategies (e.g., Wang & Hall 2021). Combining temporary suppression and long-term cognitive reappraisal to deal with adverse classroom emotions and instructional situations may in fact be most optimal in supporting students and teachers in capitalizing on both the short- and long-term benefits of these approaches (Wang & Burić, 2023; Wang et al., 2022. Our findings thus underscore the importance of understanding the nuances of teacher anger and how daily classroom dynamics can affect how teachers’ expressions of anger impact students. In contrast to describing emotional labor strategies as simply “adaptive” or “maladaptive,” this research highlights how multiple factors, including teacher traits, their relationships with students, as well as day-to-day changes in instructional contexts, determine whether such strategies are beneficial, ineffective, or harmful in a classroom setting.

## Data Availability

The datasets generated during and/or analyzed during the current study are available from the corresponding author upon reasonable request.

## Footnotes

1 This sample size exceeds the minimum recommended sample of 440 for a structural equation model that accounts for 16% of the variance (Wolf et al., 2013).

2 Single items were assessed for each emotional labor strategy, and to assess each dimension of perceived student engagement, to minimize time and fatigue commonly experienced by participants in diary studies (Ohly et al., 2010).

3 We further tested the reverse paths concerning whether the prior day’s emotional engagement (-1) or behavioral engagement (-1) were significantly linked to expressed anger, hiding anger, or faking anger. The SEM analysis showed none of the paths to be statistically significant.

## References

[CR1] Assor A, Kaplan H, Kanat-Maymon Y, Roth G (2005). Directly controlling teacher behaviors as predictors of poor motivation and engagement in girls and boys: The role of anger and anxiety. Learning and Instruction.

[CR2] Beach R, Pearson D (1998). Changes in preservice teachers’ perceptions of conflicts and tensions. Teaching and Teacher Education.

[CR3] Beal, D. J., & Trougakos, J. P. (2013). Episodic intrapersonal emotion regulation: Or, dealing with life as it happens. In A. A. Grandey, J. M. Diefendorff, & D. E. Rupp (Eds.), *Emotional labor in the 21st century: Diverse perspectives on emotion regulation at work* (pp. 31–55). Routledge/Taylor & Francis Group.

[CR5] Becker ES, Goetz T, Morger V, Ranellucci J (2014). The importance of teachers’ emotions and instructional behavior for their students’ emotions – an experience sampling analysis. Teaching and Teacher Education.

[CR4] Becker ES, Keller MM, Goetz T, Frenzel AC, Taxer JL (2015). Antecedents of teachers’ emotions in the classroom: An intraindividual approach. Frontiers in Psychology.

[CR6] Benjamini Y, Krieger AM, Yekutieli D (2006). Adaptive linear step-up procedures that control the false discovery rate. Miometrika.

[CR7] Berkowitz L, Harmon-Jones E (2004). Toward an understanding of the determinants of anger. Emotion.

[CR8] Bertsekas, D. P. (2014). *Constrained optimization and lagrange multiplier methods*. Academic Press.

[CR9] Bibou-Nakou I, Kiosseoglou G, Stogiannidou A (2000). Elementary teachers’ perceptions regarding school behavior problems: Implications for school psychological services. Psychology in the Schools.

[CR10] Blömeke S, Jentsch A, Ross N, Kaiser G, König J (2022). Opening up the black box: Teacher competence, instructional quality, and students’ learning progress. Learning and Instruction.

[CR11] Brotheridge CM, Grandey AA (2002). Emotional labor and burnout: Comparing two perspectives of “people work. Journal of Vocational Behavior.

[CR12] Brotheridge CM, Lee RT (2003). Development and validation of the emotional labour scale. Journal of Occupational and Organizational Psychology.

[CR13] Burić I, Frenzel AC (2019). Teacher anger: New empirical insights using a multi-method approach. Teaching and Teacher Education.

[CR14] Burić, I., & Frenzel, A. C. (2020). Teacher emotional labour, instructional strategies, and students’ academic engagement: A multilevel analysis. *Teachers and Teaching*, 1–18. 10.1080/13540602.2020.1740194.

[CR15] Burić I, Slišković A, Penezić Z (2019). Understanding teacher well-being: A cross-lagged analysis of burnout, negative student-related emotions, psychopathological symptoms, and resilience. Educational Psychology.

[CR16] Butler R, Shibaz L (2014). Striving to connect and striving to learn: Influences of relational and mastery goals for teaching on teacher behaviors and student interest and help seeking. International Journal of Educational Research.

[CR17] Cable DM, Edwards JR (2004). Complementary and supplementary fit: A theoretical and empirical integration. Journal of Applied Psychology.

[CR18] Callahan J (2016). Encouraging retention of new teachers through mentoring strategies. Delta Kappa Gamma Bulletin.

[CR19] Carson, R. L. (2006). *Exploring the episodic nature of teachers’ emotions as it relates to teacher burnout*. Purdue University.

[CR20] Carver CS, Harmon-Jones E (2009). Anger is an approach-related affect: Evidence and implications. Psychological Bulletin.

[CR21] Cassady JC, Finch WH (2020). Revealing nuanced relationships among cognitive test anxiety, motivation, and self-regulation through curvilinear analyses. Frontiers in Psychology.

[CR23] Chang ML (2009). An appraisal perspective of teacher burnout: Examining the emotional work of teachers. Educational Psychology Review.

[CR22] Chang CF, Hall NC, Lee SY, Wang H (2022). Teachers’ social goals and classroom engagement: The mediating role of teachers’ self-efficacy. International Journal of Educational Research.

[CR24] Chen J (2019). Efficacious and positive teachers achieve more: Examining the relationship between teacher efficacy, emotions, and their practicum performance. The Asia-Pacific Education Researcher.

[CR25] Cheng BH, McCarthy JM (2018). Understanding the dark and bright sides of anxiety: A theory of workplace anxiety. Journal of Applied Psychology.

[CR26] Chiu MM, Lehmann-Willenbrock N (2016). Statistical discourse analysis: Modeling sequences of individual actions during group interactions across time. Group Dynamics: Theory Research and Practice.

[CR27] Cohen J (1988). Set correlation and contingency tables. Applied Psychological Measurement.

[CR28] Côté, S., Van Kleef, G. A., & Sy, T. (2013). The social effects of emotion regulation in organizations. In A. Grandey, J. Diefendorff, & D. E. Rupp (Eds.), *Emotional labor in the 21st Century: Diverse perspectives on emotion regulation at work* (pp. 79–100). Routledge /Taylor & Francis Group.

[CR108] de Ruiter, J. A., Poorthuis, A. M., & Koomen, H. M. (2021). Teachers’ emotional labor in response to daily events with individual students: The role of teacher?student relationship quality. *Teaching and Teacher Education*, *107*, 103467.

[CR29] Deci EL, Ryan RM (2000). The “what” and “why” of goal pursuits: Human needs and the self-determination of behavior. Psychological Inquiry.

[CR30] Emmer ET (1994). Towards an understanding of primacy of classroom management and discipline. Teaching Education.

[CR31] Eysenck MW, Calvo MG (1992). Anxiety and performance: The processing efficiency theory. Cognition and Emotion.

[CR32] Frenzel, A. C. (2014). Teacher emotions. In R. Pekrun, & L. Linnenbrink-Garcia (Eds.), *International handbook of emotions in education* (pp. 494–519). Routledge.

[CR35] Frenzel AC, Goetz T, Lüdtke O, Pekrun R, Sutton RE (2009). Emotional transmission in the classroom: Exploring the relationship between teacher and student enjoyment. Journal of Educational Psychology.

[CR36] Frenzel AC, Pekrun R, Goetz T, Daniels LM, Durksen TL, Becker-Kurz B, Klassen RM (2016). Measuring teachers’ enjoyment, anger, and anxiety: The teacher Emotions Scales (TES). Contemporary Educational Psychology.

[CR33] Frenzel AC, Becker-Kurz B, Pekrun R, Goetz T, Lüdtke O (2018). Emotion transmission in the classroom revisited: A reciprocal effects model of teacher and student enjoyment. Journal of Educational Psychology.

[CR34] Frenzel AC, Daniels L, Burić I (2021). Teacher emotions in the classroom and their implications for students. Educational Psychologist.

[CR37] Georgiou SN, Christou C, Stavrinides P, Panaoura G (2002). Teacher attributions of student failure and teacher behavior toward the failing student. Psychology in the Schools.

[CR38] Glomb TM, Tews MJ (2004). Emotional labor: A conceptualization and scale development. Journal of Vocational Behavior.

[CR39] Goetz T, Becker ES, Bieg M, Keller MM, Frenzel AC, Hall NC (2015). The glass half empty: How emotional exhaustion affects the state-trait discrepancy in self-reports of teaching emotions. PLoS One.

[CR40] Graham, S., & Williams, C. (2009). An attributional approach to motivation in school. In K. R. Wentzel & D. B. Miele (Eds.), *Handbook of motivation at school* (pp. 25–48). Routledge. 10.4324/9780203879498

[CR41] Grandey AA, Gabriel AS (2015). Emotional labor at a crossroads: Where do we go from here?. Annual Review of Organizational Psychology and Organizational Behavior.

[CR42] Grandey AA, Melloy RC (2017). The state of the heart: Emotional labor as emotion regulation reviewed and revised. Journal of Occupational Health Psychology.

[CR43] Grant AM, Schwartz B (2011). Too much of a good thing: The challenge and opportunity of the inverted U. Perspective on Psychological Science.

[CR44] Gross JJ (1998). Antecedent-and response-focused emotion regulation: Divergent consequences for experience, expression, and physiology. Journal of Personality and Social Psychology.

[CR45] Gross JJ (2015). Emotion regulation: Current status and future prospects. Psychological Inquiry.

[CR46] Hagenauer G, Volet SE (2014). I don’t hide my feelings, even though I try to”: Insight into teacher educator emotion display. The Australian Educational Researcher.

[CR47] Hagenauer G, Hascher T, Volet SE (2015). Teacher emotions in the classroom: Associations with students’ engagement, classroom discipline and the interpersonal teacher-student relationship. European Journal of Psychology of Education.

[CR48] Harmon-Jones C, Bastian B, Harmon-Jones E (2016). Detecting transient emotional responses with improved self-report measures and instructions. Emotion.

[CR49] Hatfield, E., Cacioppo, J. T., & Rapson, R. L. (1994). *Emotional contagion*. Cambridge University Press.

[CR50] Hochschild, A. R. (1983). *The managed heart: Commercialization of human feeling*. University of California Press.

[CR51] Hox, J., Moerbeek, M., & van de Schoot, R. (2017). *Multilevel analysis: Techniques and applications*. Routledge. 10.4324/9781315650982.

[CR52] Hu LT, Bentler PM (1999). Cutoff criteria for fit indexes in covariance structure analysis: Conventional criteria versus new alternatives. Structural Equation Modeling: A Multidisciplinary Journal.

[CR53] Hülsheger UR, Schewe AF (2011). On the costs and benefits of emotional labor: A meta-analysis of three decades of research. Journal of Occupational Health Psychology.

[CR54] Hülsheger UR, Lang JW, Maier GW (2010). Emotional labor, strain, and performance: Testing reciprocal relationships in a longitudinal panel study. Journal of Occupational Health Psychology.

[CR55] Jöreskog, K. G., & Sörbom, D. (2018). *LISREL 10 for Windows*. Scientific Software International, Inc. https://ssicentraldev.azurewebsites.net/index.php/product/lisrel.

[CR56] Kajs LT (2002). Framework for designing a mentoring program for novice teachers. Mentoring and Tutoring.

[CR58] Keller MM, Chang ML, Becker E, Goetz T, Frenzel AC (2014). Teachers’ emotional experiences and exhaustion as predictors of emotional labor in the classroom: An experience sampling study. Frontiers in Psychology.

[CR59] Keller, M. M., Frenzel, A. C., Goetz, T., Pekrun, R., & Hensley, L. (2014b). Exploring teacher emotions: A literature review and an experience sampling study. In P. W. Richardson, S. A. Karabenick, & H. M. G. Watt (Eds.), *Teacher motivation: Theory and practice* (pp. 69–82). Routledge.

[CR60] Keller MM, Hoy AW, Goetz T, Frenzel AC (2016). Teacher enthusiasm: Reviewing and redefining a complex construct. Educational Psychology Review.

[CR57] Keller MM, Becker ES, Frenzel AC, Taxer JL (2018). When teacher enthusiasm is authentic or inauthentic: Lesson profiles of teacher enthusiasm and relations to students’ emotions. AERA Open.

[CR61] Kennedy, P. (2008). *Guide to econometrics*. Wiley-Blackwell.

[CR62] Klusmann U, Aldrup K, Roloff J, Lüdtke O, Hamre BK (2022). Does instructional quality mediate the link between teachers’ emotional exhaustion and student outcomes? A large-scale study using teacher and student reports. Journal of Educational Psychology.

[CR63] König J, Blömeke S, Jentsch A, Schlesinger L, Musekamp F, Kaiser G (2021). The links between pedagogical competence, instructional quality, and mathematics achievement in the lower secondary classroom. Educational Studies in Mathematics.

[CR64] Kunter M, Klusmann U, Baumert J, Richter D, Voss T, Hachfeld A (2013). Professional competence of teachers: Effects on instructional quality and student development. Journal of Educational Psychology.

[CR65] Kuppens P, Verduyn P (2015). Looking at emotion regulation through the window of emotion dynamics. Psychological Inquiry.

[CR66] Lavy S, Eshet R (2018). Spiral effects of teachers’ emotions and emotion regulation strategies: Evidence from a daily diary study. Teaching and Teacher Education.

[CR67] Lazarus, R. S. (1991). *Emotion and adaptation*. Oxford University Press.

[CR68] Lazarus, R. S., & Folkman, S. (1984). *Stress, appraisal, and coping*. Springer Publishing.

[CR69] Lennard AC, Scott BA, Johnson RE (2019). Turning frowns (and smiles) upside down: A multilevel examination of surface acting positive and negative emotions on well-being. Journal of Applied Psychology.

[CR70] Lerner JS, Keltner D (2001). Fear, anger, and risk. Journal of Personality and Social Psychology.

[CR71] Little, T. D., Card, N. A., Bovaird, J. A., Preacher, K. J., & Crandall, C. S. (2012). Structural equation modeling of mediation and moderation with contextual factors. In A. Bovaird, & N. A. Card (Eds.), *Modeling contextual effects in longitudinal studies* (pp. 207–230). Routledge.

[CR72] Ljung GM, Box GE (1979). The likelihood function of stationary autoregressive-moving average models. Biometrika.

[CR73] MacKinnon DP, Lockwood CM, Williams J (2004). Multivariate Behavioral Research.

[CR74] Manstead, A. S., & Fischer, A. H. (2001). Social appraisal. In K. R. Scherer, A. Schorr, & T. Johnstone (Eds.), *Appraisal process in emotion* (pp. 221–232). Oxford University Press.

[CR110] Ohly, S., Sonnentag, S., Niessen, C., & Zapf, D. (2010). Diary studies in organizational research. *Journal of Personnel Psychology*, *9*, 79–93. https://doi.org/10.1027/1866-5888/a000009

[CR75] Parkinson B, Manstead AS (2015). Current emotion research in social psychology: Thinking about emotions and other people. Emotion Review.

[CR76] Pekrun R (2006). The control-value theory of achievement emotions: Assumptions, corollaries, and implications for educational research and practice. Educational Psychology Review.

[CR77] Peugh JL, Enders CK (2004). Missing data in educational research: A review of reporting practices and suggestions for improvement. Review of Educational Research.

[CR78] Philipp A, Schüpbach H (2010). Longitudinal effects of emotional labour on emotional exhaustion and dedication of teachers. Journal of Occupational Health Psychology.

[CR79] Ripski MB, LoCasale-Crouch J, Decker L (2011). Pre-service teachers: Dispositional traits, emotional states, and quality of teacher-student interactions. Teacher Education Quarterly.

[CR80] Rivers SE, Brackett MA, Katulak NA, Salovey P (2007). Regulating anger and sadness: An exploration of discrete emotions in emotion regulation. Journal of Happiness Studies.

[CR81] Schiefele U (2017). Classroom management and mastery-oriented instruction as mediators of the effects of teacher motivation on student motivation. Teaching and Teacher Education.

[CR82] Skinner EA, Kindermann TA, Furrer CJ (2009). A motivational perspective on engagement and disaffection: Conceptualization and assessment of children’s behavioral and emotional participation in academic activities in the classroom. Educational and Psychological Measurement.

[CR83] Sonnentag S, Starzyk A (2015). Perceived prosocial impact, perceived situational constraints, and proactive work behavior: Looking at two distinct affective pathways. Journal of Organizational Behavior.

[CR84] Sutton RE (2004). Emotional regulation goals and strategies of teachers. Social Psychology of Education.

[CR85] Sutton, R. E. (2007). Teachers’ anger, frustration, and self-regulation. In P. A. Schutz & R. Pekrun (Eds.), *Emotion in Education* (pp. 259–274). Elsevier Academic Press. 10.1016/B978-012372545-5/50016-2

[CR87] Sutton RE, Wheatley KF (2003). Teachers’ emotions and teaching: A review of the literature and directions for future research. Educational Psychology Review.

[CR86] Sutton RE, Mudrey-Camino R, Knight CC (2009). Teachers’ emotion regulation and classroom management. Theory into Practice.

[CR88] Taxer JL, Frenzel AC (2015). Facets of teachers’ emotional lives: A quantitative investigation of teachers’ genuine, faked, and hidden emotions. Teaching and Teacher Education.

[CR89] Taxer JL, Frenzel AC (2018). Inauthentic expressions of enthusiasm: Exploring the cost of emotional dissonance in teachers. Learning and Instruction.

[CR90] Taxer JL, Gross JJ (2018). Emotion regulation in teachers: The “why” and “how. Teaching and Teacher Education.

[CR91] Van Kleef GA (2009). How emotions regulate social life: The emotions as social information (EASI) model. Current Directions in Psychological Science.

[CR92] Villar A, Strong M (2007). Is mentoring worth the money? A benefit-cost analysis and five-year rate of return of a comprehensive mentoring program for beginning teachers. ERS Spectrum.

[CR93] Wang H, Hall NC (2018). A systematic review of teachers’ causal attributions: Prevalence, correlates, and consequences. Frontiers in Psychology.

[CR94] Wang H, Hall NC (2019). When “I care” is not enough: An interactional analysis of teacher values, value congruence, and well-being. Teaching and Teacher Education.

[CR95] Wang H, Hall NC (2021). Exploring relations between teacher emotions, coping strategies, and intentions to quit: A longitudinal analysis. Journal of School Psychology.

[CR99] Wang H, Hall NC, Goetz T, Frenzel AC (2017). Teachers’ goal orientations: Effects on classroom goal structures and emotions. British Journal of Educational Psychology.

[CR97] Wang H, Hall NC, Taxer JL (2019). Antecedents and consequences of teachers’ emotional labor: A systematic review and meta-analytic investigation. Educational Psychology Review.

[CR98] Wang H, Hall NC, Chiu MM, Goetz T, Gogol K (2020). Exploring the structure of teachers’ emotional labor in the classroom: A multitrait–multimethod analysis. Educational Measurement: Issues and Practice.

[CR96] Wang H, Hall NC, King RB (2021). A longitudinal investigation of teachers’ emotional labor, well-being, and perceived student engagement. Educational Psychology.

[CR100] Wang H, Lee SY, Hall NC (2022). Coping profiles among teachers: Implications for emotions, job satisfaction, burnout, and quitting intentions. Contemporary Educational Psychology.

[CR101] Watt HM, Richardson PW (2007). Motivational factors influencing teaching as a career choice: Development and validation of the FIT-choice scale. Journal of Experimental Education.

[CR102] Watt HM, Richardson PW (2008). Motivations, perceptions, and aspirations concerning teaching as career for different types of beginning teachers. Learning and Instruction.

[CR103] Weiner B (2000). Intrapersonal and interpersonal theories of motivation from an attributional perspective. Educational Psychology Review.

[CR104] Weiner B (2010). The development of an attribution-based theory of motivation: A history of ideas. Educational Psychologist.

[CR106] Wilkowski BM, Robinson MD, Troop-Gordon W (2010). How does cognitive control reduce anger and aggression? The role of conflict monitoring and forgiveness processes. Journal of Personality and Social Psychology.

[CR109] Wolf, E. J., Harrington, K. M., Clark, S. L., & Miller, M. W. (2021). Sample size requirements for structural equation models: An evaluation of power, bias, and solution propriety. *Educational and Psychological Measurement*, *73*(6), 913–934. 10.1177/0013164413495237. 10.1177/0013164413495237 PMC4334479 25705052

[CR107] Yin H, Huang S, Chen G (2019). The relationships between teachers’ emotional labor and their burnout and satisfaction: A meta-analytic review. Educational Research Review.

